# B7-H4 Polymorphism Influences the Prevalence of Diabetes Mellitus and Pro-Atherogenic Dyslipidemia in Patients with Psoriasis

**DOI:** 10.3390/jcm11216235

**Published:** 2022-10-22

**Authors:** Wenjing Yang, Qiong Huang, Ling Han, Bing Wang, Nikhil Yawalkar, Zhenghua Zhang, Kexiang Yan

**Affiliations:** 1 Department of Dermatology, Huashan Hospital, Fudan University, Shanghai 200040, China; 2 Department of Dermatology, Inselspital, Bern University Hospital, University of Bern, 3010 Bern, Switzerland

**Keywords:** psoriasis, diabetes mellitus, B7-H4, dyslipidemia, polymorphism, methotrexate

## Abstract

Background: The co-inhibitory molecule B7-H4 is located in the genomic regions associated with type 1 diabetes (T1D) susceptibility. However, the correlation of B7-H4 with glycometabolism and dyslipidemia has never been studied. Objective: To explore the influence of B7-H4 polymorphism on the prevalence of diabetes mellitus (DM) and dyslipidemia in psoriasis. Methods: In this single-center cross-sectional study, we recruited 265 psoriatic patients receiving methotrexate (MTX) treatment. Thirteen single-nucleotide polymorphisms (SNPs) in B7-H4 were genotyped. Serum levels of total cholesterol (TC), triglycerides (TG), lipoprotein (a) (LP(a)), high-density lipoprotein cholesterol (HDL-C), low-density lipoprotein (LDL), apolipoprotein A1 (ApoA1), and apolipoprotein B (ApoB) were measured at baseline and week 12. Results: The GG genotype carriers of *rs12025144* in B7-H4 had a higher prevalence of DM (57.14% vs. 17.71% vs. 18.67%, *p* = 0.0018), and had a poorer response to MTX in diabetic patients (*p* < 0.05), compared with AA or AG genotype carriers. The AG genotype of *rs2066398* was associated with higher levels of pro-atherogenic lipids. MTX significantly downregulated the level of anti-atherogenic lipid ApoA1 in AA genotype carriers of *rs2066398*. Conclusions: The genotypes *rs12025144* and *rs2066398* in B7-H4 were correlated with a higher prevalence of DM and dyslipidemia in psoriasis, respectively.

## 1. Introduction

Psoriasis is a chronic, inflammatory skin disease mediated by interactions between keratinocytes, dendritic cells, and distinct T cells (Th1 and primarily Th17 cells) [1]. B7-H4 is a newly discovered B7 family member that negatively regulates T-cell immunity, and it is located in the genomic regions associated with type 1 diabetes (T1D). Moreover, B7-H4 has been shown to protect islet β cells against attack by Th1 cells [2], Th17 cells [3], and self-reactive CD8+ T cells [4] by co-localizing with insulin on islet β cells [5]. Although B7-H4 negatively regulates Th1 and Th17 cell responses, the role of B7-H4 in psoriasis and its comorbidities have yet to be elucidated.

Diabetes mellitus (DM) is a category of metabolic illnesses defined by chronic hyperglycemia resulting from an absolute or relative insulin shortage and insulin resistance (IR). Psoriatic patients with type 2 diabetes (T2D) had a significantly higher IR than T2D patients without psoriasis. However, psoriatic patients with T1D may have relatively preserved pancreatic β cell function due to their significantly higher levels of C-peptide in both the fasted state and after the glucose challenge [6]. Epidemiological studies report that psoriasis is associated with increased incidence rates of new-onset DM, and the severity of psoriasis is correlated with the risk of T2D [7]. Notably, psoriasis and T2D share genetic and pathological similarities as well as common targets, such as *CDKAL1*, *PSORS2*, *PSORS3*, and *PSORS4*, which are psoriasis susceptibility genes that are also associated with T2D [8].

B7-H4 is expressed on islet β cells and co-localizes with insulin; however, the effect of B7-H4 on the secretion and action of insulin has not yet been explored. In this study, we analyzed the association of the genetic variation of 13 single nucleotide polymorphisms (SNPs) in B7-H4 with the prevalence of DM and with lipid profiles in 265 psoriatic patients.

## 2. Patients and Methods

### 2.1. Patients and Study Design

A total of 265 psoriatic patients aged ≥18 years were recruited at the Department of Dermatology at Huashan Hospital, Fudan University, between February 2015 and August 2019. All participants received oral methotrexate (MTX) therapy for 12 weeks. The medical ethics committee of Huashan Hospital authorized the protocol (approval number: MTX201501), and all patients provided informed written consent. The diagnoses were based on typical clinical features and/or histopathological criteria. Excluded were those who had received systemic therapies (acitretin, cyclosporin, glucocorticoids) for arthritis or psoriasis within the previous month. The topical treatments were discontinued for a minimum of 1 week prior to the study’s onset. The therapeutic regimen followed the European guidelines on contraindications and restrictions on MTX. None of the patients used lipid-lowering drugs.

### 2.2. Treatment

The first oral dosage of MTX ranged from 7.5 to 10 mg once weekly. The dose was increased by 2.5 mg every 2–4 weeks to a maximum of 15 mg weekly, depending on the patient’s clinical response, side effects, and hematology/chemistry test results. If liver enzymes were >2- and <3-fold higher than normal, the MTX dose was lowered by 2.5 mg per week and administered once 2–4 weeks later. MTX medication was terminated if liver enzyme levels were raised by more than 3-fold [9].

### 2.3. Assessment of Lipid Profiles and Disease Characteristics

Two licensed dermatologists used the psoriasis area severity index (PASI) and body surface area (BSA) score to assess the severity and extent of psoriasis. Standard laboratory procedures at Huashan Hospital were used to evaluate lipid profiles at baseline and after 12 weeks of MTX therapy, as well as fasting blood glucose at baseline. The following were recorded: sex, age, age at disease onset, smoking habits, alcohol intake, hypertension, diabetes, height, weight, and body mass index (BMI).

### 2.4. DNA Extraction and Genotyping Analysis

A total of 5 mL of EDTA-anticoagulated whole blood was collected from each patient and stored at −80 °C. Genomic DNA was extracted from peripheral blood lymphocytes using the FlexiGene DNA Purification Kit (Qiagen, Hilden, Germany) and diluted to 20 ng/µL. All DNA samples were stored at –20 °C. The 13 SNPs of B7-H4 (*rs1935780*, *rs12034996*, *rs10047089*, *rs12025144*, *rs6674646*, *rs10923228*, *rs10923229*, *rs10754339*, *rs2358817*, *rs7524748*, *rs6657810*, *rs2066398*, *rs3738414*) were genotyped using a SequenomMassARRAY.

The SequenomMassARRAY Assay Design 3.0 software was used to design the PCR parameters and detection primers. The PCR products were subsequently used as templates for locus-specific single-base extension reactions. The resulting products were desalted and transferred to a 384-element SpectroCHIP array (Sequenom Inc., San Diego, CA, USA). MALDI-TOF MS (Sequenom Inc.) was used for allele detection. The mass spectrograms were analyzed using MassARRAY software (Sequenom Inc.). We performed quality control of SNPs and samples at a call rate of 99.7%, and we analyzed the distribution of 13 SNPs in psoriasis patients with the Hardy–Weinberg equilibrium (*p* > 0.05).

### 2.5. Statistical Analysis

The data are expressed as mean ± standard deviation (SD). As applicable, statistical analyses were conducted using the Mann–Whitney *U* test, unpaired *t*-test, unpaired *t*-test with Welch correction, paired *t*-test, χ^2^ test, or Fisher’s exact test. After adjusting for sex, age, age at disease onset, disease duration, height, weight, BMI, hypertension, diabetes, smoking, and baseline PASI score, a stepwise multiple regression analysis was performed. GraphPad Prism version 8 (Software Inc., La Jolla, CA, USA) and SPSS Statistics version 23.0 (IBM Inc., Chicago, IL, USA) software were utilized for data analysis. A *p*-value less than 0.05 was regarded as statistically significant.

## 3. Results

### 3.1. Association between GG Genotype of rs12025144 in B7-H4 and Prevalence of DM in Psoriatic Patients

The SNP *rs12025144* in B7-H4 was successfully genotyped in 264 of 265 psoriatic patients. Patients’ clinical characteristics and lipid levels are summarized in Table 1. The prevalence of DM in GG genotype carriers of *rs12025144* was significantly higher than in AA and AG genotype carriers (57.14% vs. 17.71% vs. 18.67%, *p* = 0.0018). Furthermore, GG genotype carriers of *rs12025144* had an older age, older age at disease onset, higher weight and BMI, shorter disease duration, and lower prevalence of hypertension compared to AA and AG genotype carriers; however, no statistical difference was found due to the small sample size of patients with the GG genotype. Univariate regression analysis demonstrated that age (*p* = 0.000), age at disease onset (*p* = 0.001), disease duration (*p* = 0.011), weight (*p* = 0.015), BMI (*p* = 0.004), and hypertension (*p* = 0.001) were positively correlated with the prevalence of DM in psoriatic patients, as well as with the genotypes of *rs12025144*, *rs12034996*, and *rs10047089*. Only age (*p* = 0.000), BMI (*p* = 0.004), and the *rs12025144* genotype (*p* = 0.004) were significantly correlated with DM in psoriatic patients in multivariate analysis (Table 2).

### 3.2. Association between AG Genotype of rs2066398 in B7-H4 and Levels of Pro-Atherogenic Lipids

The SNP *rs2066398* in B7-H4 was successfully genotyped in 263 of 265 psoriatic patients. As shown in Table 1, the differences in clinical characteristics and lipid levels were compared between psoriatic patients with the AA or AG genotype of *rs2066398*. Our results demonstrated that AG genotype carriers of *rs2066398* had significantly higher levels of the pro-atherogenic lipids TC (5.07 ± 1.11 vs. 4.71 ± 0.88, *p* = 0.0169), LDL (3.21 ± 0.93 vs. 2.93 ± 0.77, *p* = 0.0302), ApoB (0.81 ± 0.21 vs. 0.74 ± 0.16, *p* = 0.0092), and lower mean MTX cumulative dosage (127.8 ± 23.90 vs. 137.3 ± 20.51, *p* = 0.0059). However, no significant differences were found in clinical characteristics at baseline. Univariate regression analysis showed that age (*p* = 0.001), age at disease onset (*p* = 0.003), weight (*p* = 0.000), BMI (*p* = 0.000), diabetes (*p* = 0.001), arthritis (*p* = 0.009), hypertension (*p* = 0.001), and the SNPs *rs2066398* (*p* = 0.009), *rs2358817* (*p* = 0.044), and *rs7524748* (*p* = 0.021) were significantly associated with the level of ApoB, but only age (*p* = 0.025), BMI (*p* = 0.001), diabetes (*p* = 0.046), and the SNP *rs2066398* (*p* = 0.012) were significantly correlated with the level of ApoB in a multiple regression analysis. In addition, a univariate regression analysis revealed that age (*p* = 0.033), weight (*p* = 0.013), BMI (*p* = 0.044), diabetes (*p* = 0.002), and the SNP *rs2066398* (*p* = 0.029) were significantly associated with the level of LDL, but only diabetes (*p* = 0.002) and the *rs2066398* SNP (*p* = 0.024) were significantly correlated with the level of LDL following multiple regression analysis. Furthermore, univariate regression analysis demonstrated that age (*p* = 0.000), age at disease onset (*p* = 0.014), diabetes (*p* = 0.007), arthritis (*p* = 0.037), and the SNPs *rs2066398* (*p* = 0.015), *rs7524748* (*p* = 0.028), and *rs2358817* (*p* = 0.044) were significantly associated with the level of TC, but only age (*p* = 0.000) and *rs2066398* (*p* = 0.021) were significantly correlated with the level of TC following multiple regression analysis (Table 3).

### 3.3. Diabetic Patients with GG Genotype of rs12025144 in B7-H4 Had a Poorer Response to MTX Relative to Those with AA and AG Genotypes in Psoriasis

Among the 264 psoriatic patients were 53 diabetic patients (8 patients with GG genotype and 45 patients with AA or AG genotypes). The differences were compared between the clinical characteristics and the lipid profiles between psoriatic patients with and without DM and diabetic patients with different genotypes of *rs12025144* (Table 4). Psoriatic patients with DM had a significantly older age (57.36 ± 12.04 vs. 45.39 ± 14.74 years, *p* < 0.0001), older age at disease onset (41.62 ± 16.1 vs. 32.99 ± 15.15 years, *p* = 0.0003), longer disease duration (15.93 ± 12.45 vs. 12.41 ± 9.97 years, *p* = 0.0299), higher weight (71.96 ± 11.74 vs. 68.07 ± 12.5 kg, *p* = 0.0439) and BMI (25.65 ± 3.09 vs. 24.04 ± 3.57, *p* = 0.0033), higher prevalence of hypertension (58.49% vs. 35.38%, *p* = 0.0028), and higher levels of TC (5.07 ± 1.06 vs. 4.69 ± 0.89, *p* = 0.0068), LDL (3.28 ± 0.86 vs. 2.90 ± 0.77, *p* = 0.0019), ApoB (0.82 ± 0.19 vs. 0.73 ± 0.16, *p* = 0.0011), and TG (1.89 ± 1.18 vs. 1.54 ± 0.87, *p* = 0.0142) than those without DM. Diabetic patients with the GG genotype of *rs12025144* had a significantly lower mean PASI improvement at week 12 compared with those with the AA or AG genotypes (43.75 ± 39.6 vs. 67.07 ± 26.94, *p* = 0.0411). Diabetic patients with the GG genotype also had shorter disease duration (10.5 ± 7.76 vs. 16.89 ± 12.93 years, *p* = 0.1831), higher weight (78.13 ± 17.72 vs. 70.84 ± 10.2 kg, *p* = 0.1072) and BMI (27.54 ± 4.18 vs. 25.3 ± 2.77, *p* = 0.0583), and a lower prevalence of hypertension (25.00% vs. 64.44%, *p* = 0.0544) than those with the AA or AG genotypes, but no statistically significant differences were found due to the small sample size of diabetic patients with the GG genotype.

### 3.4. MTX Significantly Downregulated ApoA1 in AA Genotype Carriers of rs2066398

We analyzed the effect of a 12-week MTX treatment on the lipid profiles according to the genotype of *rs2066398* in 263 psoriatic patients. As shown in Table 5, the levels of TC (*p* < 0.0001), LDL (*p* = 0.0025), ApoB (*p* < 0.0001), and Lp(a) (*p* < 0.0001) were significantly downregulated in 263 patients. However, the significant downregulation of MTX on the level of ApoA1 (1.05 ± 0.17 vs. 1.03 ± 0.16 g/L, *p* = 0.0131) was observed in the AA—but not the AG—genotype carriers of *rs2066398*.

## 4. Discussion

Our results demonstrated that the prevalence of DM in psoriatic patients with the GG genotype of *rs12025144* in B7-H4 was significantly higher than those with either the AA or AG genotype (57.14% vs. 17.71% vs. 18.67%, *p* = 0.0018). Moreover, GG genotype carriers had older age, older age at disease onset, shorter disease duration, and a higher weight and BMI relative to AA and AG genotypes carriers, but no statistically significant differences were found due to the limited sample size. According to a meta-analysis, the prevalence of DM in Afghanistan was 12.13%, and univariate meta-regression revealed that the prevalence of DM rose with age, hypertension, and obesity [10]. Other studies have also found a link between DM and the severity of psoriasis [11]. Although severe forms of psoriasis are more prevalent in males than in females, our study demonstrated that there were more women, but not more severe psoriasis, among GG genotype carriers. Our results align with those of a previous report, in which higher BMI and weight gain were found to be risk factors for incident psoriasis in older women [12,13]. Therefore, we infer that metabolic abnormalities in patients with the GG genotype may occur before the onset of psoriasis.

In addition, we further analyzed the differences in clinical characteristics in psoriatic patients with DM between the AA/AG and GG genotypes. A significantly lower mean PASI improvement at 12 weeks was observed in GG than in AA or AG genotype carriers (43.75% vs. 67.07%, *p* < 0.05), which was consistent with our previous finding that higher BMI was associated with a poorer response to MTX [14]. The GG genotype carriers with DM had a shorter disease duration and less hypertension than AA and AG genotype carriers with DM, which differed from the finding that psoriatic patients with DM had a significantly higher prevalence rate of hypertension (58.49% vs. 35.38%, *p* = 0.0028) and longer disease duration (15.93 ± 12.45 vs. 12.41 ± 9.97 years, *p* = 0.0299) compared with those without DM. Furthermore, significantly increased levels of the pro-atherogenic lipids TC, TG, LDL, and ApoB were found. Another study also reported higher levels of TC and LDL in psoriatic patients with DM than in those without DM [15].

The human B7-H4 gene (VTCN1) is found on chromosome 1p13.1 [16], which is among the genomic regions linked to T1D susceptibility (insulin-dependent diabetes gene: Idd10) [17,18]. T1D is characterized by T-cell responses directed against insulin-secreting pancreatic β cells. The reduction of membrane-tethered VTCN1 (mVTCN1) on islet cells and islet-resident macrophages has been reported to be correlated with the elevated serum soluble VTCN1 (sVTCN1) levels seen in T1D patients. Moreover, a gradual loss of mVTCN1 on the islet cells and islet-resident macrophages was mediated by metalloproteinase nardilysin (NRD1), and sVTCN1 was considerably less effective than mVTCN1 at inhibiting T-cell proliferation [19]. In addition, B7-H4 was co-localized with insulin on islet β cells [5], indicating that the B7-H4 pathway might play an important role in β cell function maintenance and insulin secretion. Therefore, we inferred that the increased rate of DM in GG genotype carriers of *rs12025144* could be mediated by the insulin-dependent diabetes gene; however, its exact role requires further exploration.

Our study also found that the AG genotype of *rs2066398* in B7-H4 was significantly associated with higher levels of pro-atherogenic lipids following multiple regression analysis. MTX significantly decreased the levels of pro-atherogenic lipids in all psoriatic patients, which corroborates the findings of a previous report [20]. However, lowering anti-atherogenic lipid levels using MTX was controversial. Our study showed that the significant downregulation induced by MTX on ApoA1 levels was only observed in AA genotype carriers of *rs2066398*. Another study also reported that MTX slightly decreased HDL levels [21]. According to reports, MTX has an anti-lipolytic effect mediated by the adenosine release [22]. Ex vivo epididymal adipose tissue from MTX-treated mice secreted less glycerol when cultured [22]. In addition, MTX promoted glucose uptake and lipid oxidation in skeletal muscle via the AMP-activated kinase (AMPK) activation [23]. Our previous study also identified that MTX could restore the immunosuppressive function of Tregs by upregulating CD73, thereby activating AMPK and inhibiting the mTOR pathway [24]. AMPK is a central regulator of energy metabolism, directly connecting MTX and energy metabolism.

Our study was limited by the lack of consensus on the classification of DM, as the registries do not hold data on, for example, HbA1C (glycosylated hemoglobin) or C-peptide levels.

In conclusion, this study firstly explores the association between B7-H4 SNPs and the prevalence of DM and pro-atherogenic dyslipidemia in psoriatic patients. The GG genotype of *rs12025144* was significantly correlated with a higher prevalence of DM in psoriatic patients. The genetic background of abnormal lipid metabolism in GG genotype carriers of *rs12025144* facilitated the induction of psoriasis. They differed from patients with psoriasis-induced DM in clinical characteristics as they had shorter psoriasis disease duration and a lower prevalence of hypertension. The AG genotype of *rs2066398* was significantly correlated with increased levels of pro-atherogenic lipids, whereas the AA genotype of *rs2066398* was correlated with the significant MTX-induced reduction in the level of the anti-atherogenic lipid ApoA1.

## 5. Conclusions

Briefly, B7-H4 may play a crucial role in abnormal glucose and lipid metabolism in psoriatic patients by regulating islet β cell function and insulin secretion.

## Figures and Tables

**Table 1:** Clinical characteristics and lipid profiles according to the *rs12025144* and *rs2066398* genotypes in psoriatic patients.

	*rs12025144*				*rs2066398*		
	AA (*n* = 175)	AG (*n* = 75)	GG (*n* = 14)	*p*-Value	AA (*n* = 217)	AG (*n* = 46)	*p*-Value
Age, y	47.85 ± 15.26	47.29 ± 14.62	49.21 ± 15.18	0.9014	47.35 ± 14.39	49.3 ± 17.73	0.4224
Age at disease onset, y	33.78 ± 15.48	35.79 ± 16.14	39.25 ± 15.73	0.3456	33.9 ± 14.9	37.59 ± 18.61	0.147
Disease duration, y	14.13 ± 11.41	11.49 ± 8.42	9.96 ± 8.76	0.0994	13.49 ± 10.57	11.71 ± 10.68	0.3017
Weight, kg	68.13 ± 12.43	69.63 ± 11.63	72.92 ± 15.9	0.3252	68.57 ± 12.24	70.1 ± 13.31	0.4572
BMI, kg/m^2^	24.2 ± 3.53	24.45 ± 3.29	26.02 ± 4.65	0.2433	24.28 ± 3.50	24.78 ± 3.73	0.3898
PASI score at baseline	14.62 ± 7.98	12.86 ± 6.78	13.29 ± 5.93	0.1412	14.28 ± 7.68	13.13 ± 7.09	0.3543
The mean PASI improvement at 12 weeks	66.09 ± 31.17	57.55 ± 32.44	58.86 ± 36.46	0.6982	63.69 ± 30.46	61.07 ± 38.73	0.6141
MTX cumulative dosage, mg	135.6 ± 21.13	136.1 ± 22.18	133.6 ± 21.07	0.0824	137.3 ± 20.51	127.8 ± 23.90	**0.0059**
Male, *n* (%)	120 (68.57)	57 (76.0)	9 (64.29)	0.385	154 (70.97)	32(69.57)	0.8595
Arthritis, *n* (%)	96 (54.86)	36 (48.0)	7 (50.0)	0.5969	114 (52.53)	24 (52.17)	>0.99
Hypertension, *n* (%)	68 (38.86)	34 (45.33)	4 (28.57)	0.4187	84 (38.71)	22 (47.83)	0.3208
Diabetes, *n* (%)	31 (17.71)	14 (18.67)	8 (57.14)	**0.0018**	44 (20.28)	9 (19.57)	>0.99
Smoking, *n* (%)	56 (32.0)	19 (25.33)	7 (50.0)	0.1681	72 (33.18)	10 (21.74)	0.1612
TC, mmol/L	4.80 ± 0.86	4.63 ± 1.05	5.11 ± 1.10	0.1541	4.71 ± 0.88	5.07 ± 1.11	**0.0169**
TG, mmol/L	1.61 ± 0.95	1.55 ± 0.88	1.89 ± 1.27	0.4734	1.61 ± 0.97	1.62 ± 0.88	0.9364
HDL-C, mmol/L	1.20 ± 0.29	1.15 ± 0.28	1.12 ± 0.33	0.3722	1.18 ± 0.27	1.20 ± 0.38	0.5816
LDL, mmol/L	3.00 ± 0.75	2.88 ± 0.89	3.16 ± 1.05	0.396	2.93 ± 0.77	3.21 ± 0.93	**0.0302**
ApoA1, g/L	1.06 ± 0.18	1.03 ± 0.17	1.05 ± 0.22	0.5528	1.05 ± 0.17	1.05 ± 0.21	0.9234
ApoB, g/L	0.75 ± 0.16	0.74 ± 0.18	0.82 ± 0.22	0.2491	0.74 ± 0.16	0.81 ± 0.21	**0.0092**
Lp(a), mg/L	148.5 ± 166.2	173.9 ± 195	108 ± 105.9	0.339	155.9 ± 176.2	143.5 ± 157.8	0.6593

ApoA1, apolipoprotein A1; ApoB, apolipoprotein B; BMI, body mass index (calculated as weight in kilograms divided by height in meters squared); HDL-C, high-density lipoprotein cholesterol; LDL: low-density lipoprotein; Lp(a), lipoprotein A; MTX, methotrexate; PASI, Psoriasis Area Severity Index; TC, total cholesterol; TG, triglyceride; y, years.

**Table 2:** Univariate and multivariate analyses of factors associated with the prevalence of diabetes mellitus in psoriatic patients.

	Univariate Analysis		Multivariate Analysis	
Predictor	OR (95% CI)	*p*-Value	OR (95% CI)	*p*-Value
Age	1.058 (1.036–1.081)	**0.000**	1.069 (1.043–1.096))	**0.000**
BMI	1.122 (1.037–1.213)	**0.004**	1.15 (1.046–1.264)	**0.004**
*rs12025144*		**0.003**		**0.004**
*rs12025144(1)*	0.153 (0.051–0.455)	**0.001**	0.115 (0.032–0.414)	**0.001**
*rs12025144(2)*	0.164 (0.051–0.525)	**0.002**	0.13 (0.034–0.499)	**0.003**
Disease duration	1.032 (1.007–1.058)	**0.011**		
Weight	1.028 (1.005–1.051)	**0.015**		
Age at disease onset	1.031 (1.013–1.049)	**0.001**		
Hypertension	2.649 (1.515–4.634)	**0.001**		
*rs12034996*		**0.018**		
*rs12034996(1)*	0.152 (0.041–0.565)	**0.005**		
*rs12034996(2)*	0.19 (0.049–0.742)	**0.017**		
*rs10047089*		**0.004**		
*rs10047089(1)*	0.171 (0.06–0.489)	**0.001**		
*rs10047089(2)*	0.211 (0.07–0.641)	**0.006**		

BMI, body mass index (calculated as weight in kilograms divided by height in meters squared); OR, odds ratio.

**Table 3:** Univariate and multivariate analyses of factors associated with the levels of pro-atherogenic lipids ApoB, LDL, and TC in psoriatic patients.

		Univariate Analysis		Multivariate Analysis	
Lipids	Predictors	OR (95% CI)	*p*-Value	OR (95% CI)	*p*-Value
ApoB	*rs2066398*	0.072 (0.018–0.125)	**0.009**	0.068 (0.015–0.12)	**0.012**
	Age	0.002 (0.001–0.004)	**0.001**	0.002 (0–0.003)	**0.025**
	BMI	0.011 (0.006–0.016)	**0.000**	0.009 (0.004–0.014)	**0.001**
	Diabetes	0.085 (0.034–0.135)	**0.001**	0.054 (0.001–0.107)	**0.046**
	*rs2358817*	0.053 (0.001–0.105)	**0.044**		
	*rs7524748*	0.059 (0.009–0.109)	**0.021**		
	Arthritis	0.054 (0.014–0.095)	**0.009**		
	Weight	0.003 (0.001–0.004)	**0.000**		
	Age at disease onset	0.002 (0.001–0.003)	**0.003**		
	Hypertension	0.068 (0.026–0.109)	**0.001**		
LDL	*rs2066398*	0.284 (0.029–0.539)	**0.029**	0.298 (0.04–0.556)	**0.024**
	Diabetes	0.38 (0.141–0.619)	**0.002**	0.393 (0.151–0.636)	**0.002**
	Weight	0.009 (0.002–0.016)	**0.013**		
	BMI	0.027 (0.001–0.053)	**0.044**		
	Age	0.007 (0.001–0.013)	**0.033**		
TC	*rs2066398*	0.365 (0.07–0.66)	**0.015**	0.34 (0.052–0.629)	**0.021**
	Age	0.014 (0.007–0.021)	**0.000**	0.014 (0.006–0.021)	**0.000**
	*rs7524748*	0.307 (0.033–0.581)	**0.028**		
	*rs2358817*	0.292 (0.008–0.577)	**0.044**		
	Diabetes	0.386 (0.107–0.664)	**0.007**		
	Age at disease onset	0.009 (0.002–0.016)	**0.014**		
	Arthritis	0.239 (0.014–0.463)	**0.037**		

ApoB, apolipoprotein B; BMI, body mass index (calculated as weight in kilograms divided by height in meters squared); LDL, low-density lipoprotein; TC, total cholesterol.

**Table 4:** Clinical characteristics and lipid profiles according to diabetes and the genotype of *rs12025144* in 265 psoriatic patients.

	PS without DM (*n* = 212)	PS with DM (*n* = 53)	*p*-Value	DM with GG (*n* = 8)	DM with AA or AG (*n* = 45)	*p*-Value
Age, y	45.39 ± 14.74	57.36 ± 12.04	**<0.0001**	51 ± 12.69	58.49 ± 11.7	0.1055
Age at disease onset, y	32.99 ± 15.15	41.62 ± 16.1	**0.0003**	40.5 ± 15.64	41.82 ± 16.45	0.8336
Disease duration, y	12.41 ± 9.97	15.93 ± 12.45	**0.0299**	10.5 ± 7.76	16.89 ± 12.93	0.1831
Weight, kg	68.07 ± 12.5	71.96 ± 11.74	**0.0439**	78.13 ± 17.72	70.84 ± 10.2	0.1072
BMI, kg/m^2^	24.04 ± 3.57	25.65 ± 3.09	**0.0033**	27.54 ± 4.18	25.3 ± 2.77	0.0583
PASI score at baseline	14.23 ± 7.66	13.33 ± 7.17	0.4435	11.35 ± 5.43	13.69 ± 7.43	0.4007
The mean PASI improvement at 12 weeks	63.34 ± 32.47	63.55 ± 29.93	0.9664	43.75 ± 39.6	67.07 ± 26.94	**0.0411**
Male, *n* (%)	148 (69.81)	39 (73.58)	>0.99	5(62.50)	34 (75.56)	0.6707
Arthritis, *n* (%)	109 (51.42)	31 (58.49)	0.442	3 (37.50)	28 (62.22)	0.2532
Hypertension, *n* (%)	75 (35.38)	31 (58.49)	**0.0028**	2 (25.00)	29 (64.44)	0.0544
Smoking, *n* (%)	64 (30.19)	19 (35.85)	0.5079	4 (50.00)	15 (33.33)	0.4363
TC, mmol/L	4.69 ± 0.89	5.07 ± 1.06	**0.0068**	5.44 ± 0.92	5.01 ± 1.07	0.2906
TG, mmol/L	1.54 ± 0.87	1.89 ± 1.18	**0.0142**	2.35 ± 1.53	1.81 ± 1.11	0.2362
HDL-C, mmol/L	1.19 ± 0.31	1.13 ± 0.19	0.1548	1.08 ± 0.16	1.14 ± 0.2	0.4068
LDL, mmol/L	2.90 ± 0.77	3.28 ± 0.86	**0.0019**	3.42 ± 1.16	3.25 ± 0.82	0.6286
ApoA1, g/L	1.05 ± 0.19	1.05 ± 0.14	0.8387	1.05 ± 0.12	1.05 ± 0.15	0.9819
ApoB, g/L	0.73 ± 0.16	0.82 ± 0.19	**0.0011**	0.91 ± 0.22	0.80 ± 0.18	0.1208
Lp(a), mg/L	147.7 ± 174.9	174.4 ± 161.9	0.3158	118.4 ± 137.8	184.3 ± 165.2	0.2928

ApoA1, apolipoprotein A1; ApoB, apolipoprotein B; BMI, body mass index (calculated as weight in kilograms divided by height in meters squared); HDL-C, high-density lipoprotein cholesterol; LDL: low-density lipoprotein; Lp(a), lipoprotein A; PASI; Psoriasis Area Severity Index; TC, total cholesterol; TG, triglyceride; y, years.

**Table 5:** The effect of MTX treatment on the levels of lipid profiles according to the genotype of *rs2066398* in 293 psoriatic patients.

	AA (*n* = 217)		*p*-Value	AG (*n* = 46)		*p*-Value
	Before MTX	After MTX		Before MTX	After MTX	
TC, mmol/L	4.71 ± 0.88	4.52 ± 0.89	**<0.0001**	5.07 ± 1.11	4.76 ± 0.99	**0.0019**
TG, mmol/L	1.61 ± 0.97	1.54 ± 0.98	0.2186	1.62 ± 0.88	1.53 ± 0.77	0.4372
HDL-C, mmol/L	1.18 ± 0.27	1.15 ± 0.25	0.0526	1.20 ± 0.38	1.21 ± 0.36	0.7485
LDL, mmol/L	2.93 ± 0.77	2.82 ± 0.77	**0.0025**	3.21 ± 0.93	2.98 ± 0.80	**0.0079**
ApoA1, g/L	1.05 ± 0.17	1.03 ± 0.16	**0.0131**	1.05 ± 0.21	1.05 ± 0.20	0.8845
ApoB, g/L	0.74 ± 0.16	0.70 ± 0.15	**<0.0001**	0.81 ± 0.21	0.72 ± 0.18	**0.0001**
Lp(a), mg/L	155.9 ± 176.2	138.8 ± 160.8	**<0.0001**	143.5 ± 157.8	122.4 ± 128.3	**0.037**

ApoA1, apolipoprotein A1; ApoB, apolipoprotein B; HDL-C, high-density lipoprotein cholesterol; LDL: low-density lipoprotein; Lp(a), lipoprotein A; TC, total cholesterol; TG, triglyceride. Paired *t*-test was used. *p* < 0.05 was considered statistically significant, and significant *p*-values are shown in bold font.

## Data Availability

The data that support the findings of this study are available from the corresponding author upon reasonable request.

## Abbreviations

AMPK	AMP-activated kinase
ApoA1	Apolipoprotein A1
ApoB	Apolipoprotein B
B7-H4	B7 Homolog 4
BMI	Body mass index
BSA	Body surface area
DM	Diabetes mellitus
HDL-C	High-density lipoprotein cholesterol
IR	Insulin resistance
LDL	Low-density lipoprotein
LP(a)	Lipoprotein (a)
MTX	Methotrexate
NRD1	Metalloproteinase nardilysin
PASI	Psoriasis area severity index
SNPs	Single-nucleotide polymorphisms
TC	Total cholesterol
TG	Triglyceride
T1D	Type 1 diabetes
T2D	Type 2 diabetes
VTCN1	V-set domain containing T cell activation inhibitor 1
